# A penalty on photosynthetic growth in fluctuating light

**DOI:** 10.1038/s41598-017-12923-1

**Published:** 2017-10-02

**Authors:** Percival J. Graham, Brian Nguyen, Thomas Burdyny, David Sinton

**Affiliations:** University of Toronto Mechanical and Industrial Engineering, Toronto, Canada

## Abstract

Fluctuating light is the norm for photosynthetic organisms, with a wide range of frequencies (0.00001 to 10 Hz) owing to diurnal cycles, cloud cover, canopy shifting and mixing; with broad implications for climate change, agriculture and bioproduct production. Photosynthetic growth in fluctuating light is generally considered to improve with increasing fluctuation frequency. Here we demonstrate that the regulation of photosynthesis imposes a penalty on growth in fluctuating light for frequencies in the range of 0.01 to 0.1 Hz (organisms studied: *Synechococcus elongatus* and *Chlamydomonas reinhardtii*). We provide a comprehensive sweep of frequencies and duty cycles. In addition, we develop a 2^nd^ order model that identifies the source of the penalty to be the regulation of the Calvin cycle – present at all frequencies but compensated at high frequencies by slow kinetics of RuBisCO.

## Introduction

In nature, solar flux is discontinuous, fluctuating due to the diurnal cycle, varying cloud cover, canopy shifting and circulation in bodies of water, photosynthetic organisms are therefore exposed to a diverse set of fluctuating light profiles ranging from 0.00001 to 10 Hz^1–4^. Terrestrial plants, typically experience fluctuations on the order of in the range of 0.001 to 1 Hz^1–5^ due to canopy shifting and varying cloud cover. In the case of aquatic microorganisms, cyanobacteria and eukaryotic algae, fluctuating irradiance is driven by a combination of mixing and spatial light heterogeneity. Specifically, light is most intense at the surface of a body of water, then decreases with depth due to turbidity. Mixing causes cells to travel upwards from areas of low light towards the photic zone, where light is sufficient to drive growth. In estuaries the resulting frequency is on the order of 0.02 to 0.0002 Hz. In contrast marine organisms may experience much slower variations, where crossing from the dark region to the photic zone may take days^6–8^.

Since light drives primary productivity, the ability of a photosynthetic organisms to capture fluctuating light, will impact its ability to fulfill its ecological role. As such, environmental parameters such as wind, circulation due to temperature gradients and turbidity can impact primary productivity. In terms of industrial applications, these fluctuating irradiance conditions are common in agriculture, as well as in photobioreactors. In agriculture, fluctuating light occurs due to variations in cloud cover and plant displacement. Fluctuating light in photobioreators is due to mixing in and out of the photic zone, similar in nature to shallow estuaries. Depending on reactor geometry, mixing can induce a variety of fluctuating light profiles, with frequencies ranging from 0.05–5 Hz, where frequencies over 1 Hz can improve reactor performance^9–17^.

Photosynthetic growth is composed of two major sets of reactions, the light dependent reactions and the light independent reactions. The light dependent reactions include the generation of ATP and NADPH from light and oxygen evolution. These reactions occur along a set of membrane bound proteins, which capture light energy and use it to split water, evolve oxygen and make ATP and NADPH. The light independent reactions use the products from the light dependent reaction (ATP and NADPH) to reduce carbon via the Calvin cycle. Typically, the light dependant reactions occur more rapidly than the light independent reactions^13,18,19^.

Photosynthetic growth in fluctuating light is generally considered to be bound by two extremes, a high frequency limit, where the growth rate is dictated by the time-averaged light intensity, (full-light-integration), and a low frequency limit where photosynthetic growth is equivalent to sum of the growth rate in each light condition (no-light-integration)^20–26^. At high frequencies (>1 Hz), photosynthetic growth occurs during both the light and the dark phase. High frequency fluctuations allow for continuous growth through a mismatch of reaction rates, specifically rapid light harvesting reactions and slower enzymatic reactions^13,18,19^. The mismatch between rates causes an accumulation of intermediate products during the light phase which can then be consumed during the dark phase, allowing for nearly continuous growth. As frequency decreases, the fraction of the dark period where growth continues decreases, and the resulting growth rate decays to the low frequency (no-light-integration) case, where there is no flashing light benefit. Specifically, the mismatch is between (i) the rapid light-driven generation of ATP and NADPH by the photosynthetic electron transport chain and (ii) the slower Calvin cycle^13,18,19,27^. In the case of very high frequencies, (>100 Hz), mismatches along the photosynthetic electron transport chain can also contribute to the flashing light effect^14,28,29^.

Motivated by the complexity and importance of photosynthetic growth under fluctuating light, a variety of models have been proposed, all of which predict growth rates between the established high and low frequency bounds^19–26,30,31^. These models range in complexity, but all contain a light dependent excitation step (capturing the formation of ATP and NADPH), and a light independent step (capturing the consumption of ATP and NADPH by the Calvin cycle), which allow them to capture the behaviour between the high and low frequency bounds. However, irradiance controls the degree of activation of the Calvin cycle by indirectly activating Rubisco Activase by increasing the amount of ATP and by promoting RuBP regeneration via redox sensing of the photosynthetic electron transport chain^32^. This regulation causes photosynthesis to not respond instantaneously to changing irradiance levels^1,32–38^, suggesting dynamics outside the high and low frequency bounds are likely.

Here we demonstrate a flashing light penalty where the photosynthetic growth is below the low frequency limit associated with no-light-integration. Specifically, growth is severely hampered for frequencies in the range of approximately 0.1 to 0.01 Hz, an effect not captured with current models. The penalty presented here is heavily dependent on duty cycle: small duty cycles have a substantial penalty, whereas large duty cycles are shown to have nearly no penalty. This penalty was present under photoautotrophic growth for both *Synechococcus elongatus* and *Chlamydomonas reinhardtii*. Under mixotrophic growth, where an organism can use organic carbon as energy, the penalty is heavily attenuated. We attribute this penalty to the regulation of Calvin cycle by irradiance, where after a sufficiently long period in the dark, the Calvin cycle is inactive. When exposed to light the Calvin cycle is activated, albeit with a time lag that is significant at frequencies in the range 0.1 to 0.01 Hz – a range that is germane to natural, agricultural and industrial systems^3,32,39^. By expanding an existing mechanistic model, we demonstrate that the partial activation state of the Calvin cycle is responsible for the frequency dependent penalty affecting photosynthetic organisms.

## Results and Discussion

**Figure 1:**
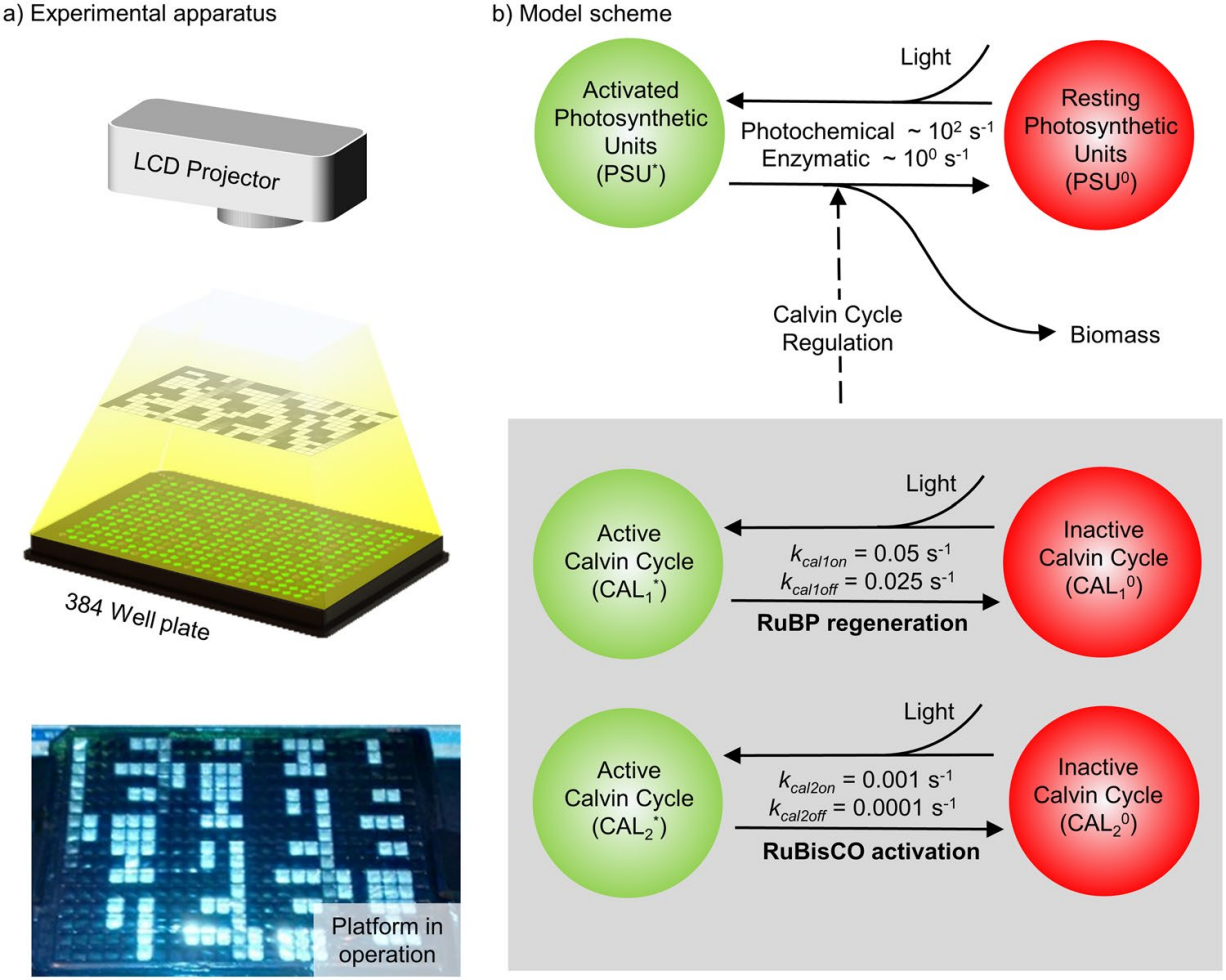
Combined experimental and analytical approach. (**a**) Schematic of irradiance technique used, consisting of a well plate aligned to a projector, with a sample image of the well plate being illuminated by the projector. (**b**) Model scheme. In the top row, photosynthetic units are excited by light, then decay to produce additional biomass. The bottom row shows regulation of the Calvin cycle by light. Specifically, the activation rates for the components of the Calvin cycle (*k*_*cal1on*_ and *k*_*cal2on*_) are the indicated values in the light and 0 in the dark. In contrast, the off rates (*k*_*cal1off*_ and *k*_*cal2off*_) are constant through the light and dark phases.

We investigate the effect of fluctuating light on photosynthetic growth through a combined experimental and analytical approach. Specifically, we investigate the effects of frequency (light-dark cycles per second) and duty cycle (fraction of a cycle where the light is at maximum intensity) on photosynthetic growth. Two organisms were chosen, *Synechococcus elongatus* and *Chlamydomonas reinhardtii*. *Synechococcus elongatus* is frequently used in biotechnology since it can be transformed for chemical production or increased growth^40,41^. Moreover the *Synechococcus* genus is present in a wide range of marine, freshwater and terrestrial environments, across a broad temperature range, thus an important contributor to primary productivity^42–44^. *Chlamydomonas reinhardtii* is a model organism where mutated strains are readily available and it typically resides in freshwater and soils^45^. In addition, Chlamydomonas reinhardtii, as a mixotroph, is capable of heterotrophic, photoheterotrophic and photoautotrophic growth. To sweep the large parameter space associated with the entire frequency spectrum relevant to photosynthetic growth and distinct duty cycles we used projection to provide independent control of light conditions in each chamber of a 384-well plate (Fig. 1a), with full details included in methods.

To gain further insight into the behaviour of photosynthetic growth in fluctuating light, we developed a mathematical model which accounts for the regulation of the Calvin cycle by irradiance (Fig. 1b). Specifically, we adapted the model presented by Camacho Rubio *et al*.^19^ to include the effect of irradiance on the degree of activation of the Calvin cycle. The degree of activation of the Calvin Cycle represents the instantaneous reaction rate relative to the rate that would be obtained under continuous irradiance. Irradiance controls the degree of activation of the Calvin cycle by indirectly activating Rubisco Activase by increasing the amount of ATP and by promoting RuBP regeneration via redox sensing of the photosynthetic electron transport chain^32^.

Both the RuBP regeneration and RuBisCO activation contribute to the degree of activation of the Calvin Cycle. In the context of the model, the degree of activation of the Calvin cycle controls the rate at which activated photosynthetic units are converted into biomass and light harvesting systems are returned to their resting state. This regulation of the reaction aligns with the role of the Calvin cycle, specifically, using energy captured by the photosynthetic electron transport to reduce carbon. The full details of the model and MATLAB scripts are included as supplementary information.

**Figure 2:**
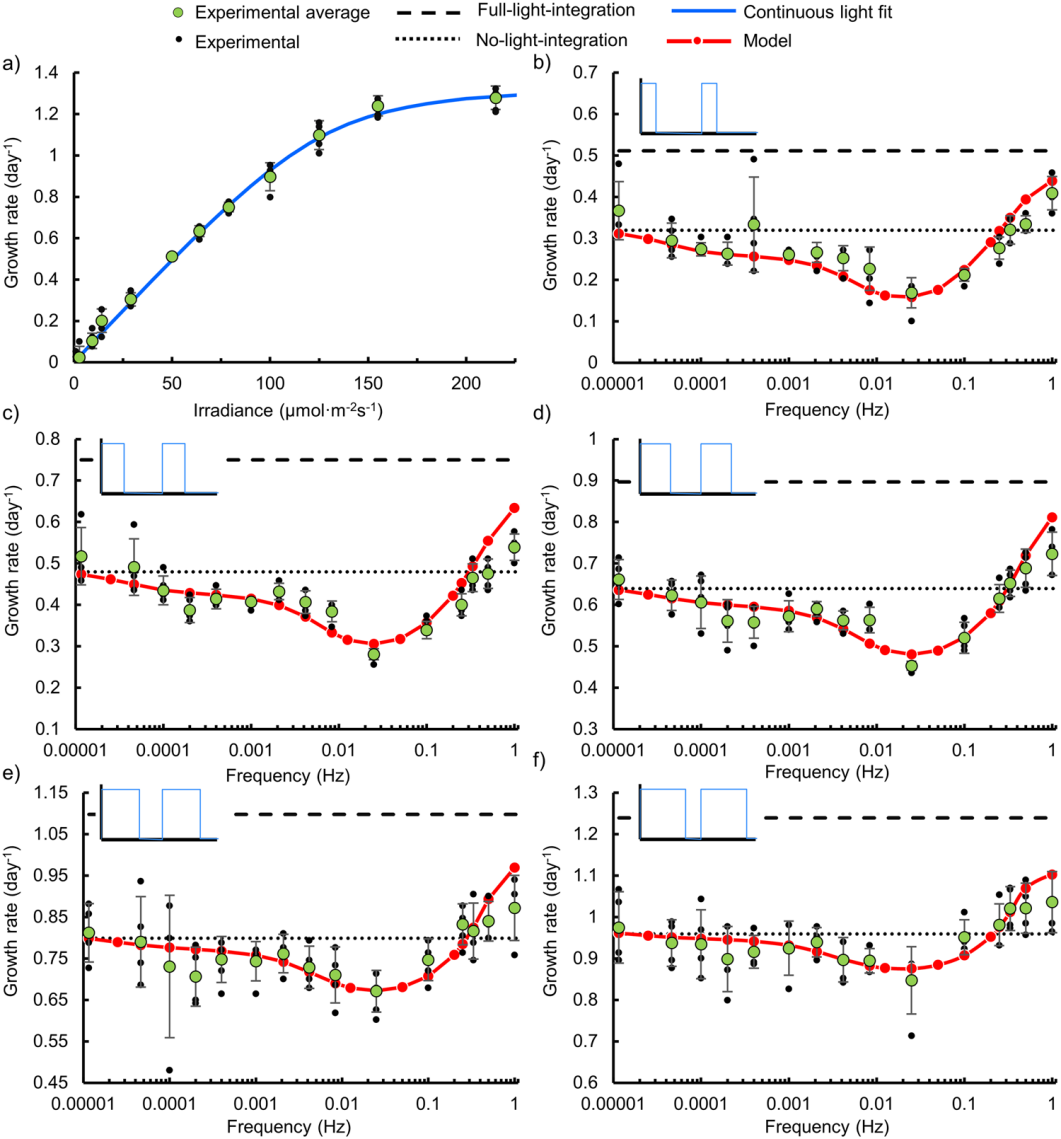
Summary of experimental data demonstrating a frequency dependent penalty for flashing light with corresponding model. (**a**) Steady state growth vs irradiance curve with the model fit to extract parameters α, κ and P_m_ (R^2^ = 0.997). The effect of frequency for various duty cycles is shown in (**b**–**f**) for square waves alternating between 215 and ≈0 µmol·m^−2^ s^−1^ with duty cycles of 0.25, 0.375, 0.5, 0.625 and 0.75 respectively. Dashed lines in (**b**–**f**) correspond full-light-integration and dotted lines correspond to no-light-integration. Experimental data corresponds to the mean and standard deviation of four data points, from a single well plate, after two days of growth, individual data points are also included.

In Fig. 2 both experimental and modelling results are presented, showing the effect of frequency and duty cycle for square wave light profiles fluctuating between an irradiance of 215 µmol·m^−2^ s^−1^ and ≈0 µmol·m^−2^ s^−1^. The frequency ranges from 0.00001 to 1 Hz for duty cycles of 0.25 (b), 0.375 (c), 0.5 (d), 0.625 (e) and 0.75 (f). The growth vs irradiance curve obtained under continuous irradiance (Fig. 2a), enables interpretation of the effect of flashing light frequency, by (i) providing the growth rate corresponding to the time averaged intensities for the five flashing light cases, (ii) demonstrating that the irradiance chosen is saturating and (iii) providing data to extract parameters to be used in the model. The parameters extracted include α, the ratio between enzymatic and photochemical rate constants, κ a half saturation constant of the Calvin cycle and Pm the maximum growth rate. The growth*-*irradiance curve shows a saturating relationship where growth increases with irradiance up until an intensity of 150 µmol·m^−2^ s^−1^, with the maximum growth rate aligning with literature values^46–48^. The growth under continuous light was obtained on the same well plate as all of the fluctuating cases. Growth curves are included as supplemental information, to demonstrate continued exponential growth over the two days (Fig. S2).

### The Calvin cycle imposes a penalty on photosynthetic growth in fluctuating light

Each frequency response plot (Fig. 2b–f) is annotated with two reference lines, (i) growth with full-light-integration, where growth would be dictated by the time-average irradiance (dashed lines in Fig. 2b–f) and (ii) growth with no-light-integration, where growth is equivalent to the sum of the growth rate in each light condition (dotted lines in Fig. 2b–f). At the extreme frequencies, our data for all duty cycles agrees with literature data - matching the high and low frequency limits^20–26^. Specifically, at high frequencies, growth approaches the full-light-integration limit. Likewise at low frequencies, growth approaches the no-light-integration limit with decreasing frequency^13,19^. However, our data shows that at frequencies ranging from 0.01 to 0.1 Hz, growth is significantly lower (Anova: Single Factor, P-values of 0.002, 0.002, 2.4 × 10^−8^, 8 × 10^−11^ and 4 × 10^−9^ for duty cycles of 0.75, 0.625, 0.5, 0.375 and 0.25 respectively, for n = 12 for all duty cycles except 0.625 where n = 8) than the low frequency case – indicating a penalty. Specifically, the minimum growth rate is as low as 50 percent of the no-light-integration case for the 0.25 duty cycle.

The presence of a penalty suggests behaviour outside the mismatch between photochemical and enzymatic reactions. We hypothesize that this penalty is caused by the regulation of the Calvin cycle by light^32,39,49^, which is strongly supported by the agreement between our model and experimental data. The parameters used in the model align with the range of rate constants presented in literature for both the activation and deactivation rates of (i) RuBP regeneration (*k*_*cal1on*_ = 0.05, *k*_*cal1off*_ = 0.025) and (ii) RuBisCO activation (*k*_*cal2on*_ = 0.001, *k*_*cal2off*_ = 0.0001)^2,8,32,39,50–57^, justifying the role of Calvin cycle regulation in the observed penalty. A full discussion regarding the parameters employed here is provided as supplemental information.

**Figure 3:**
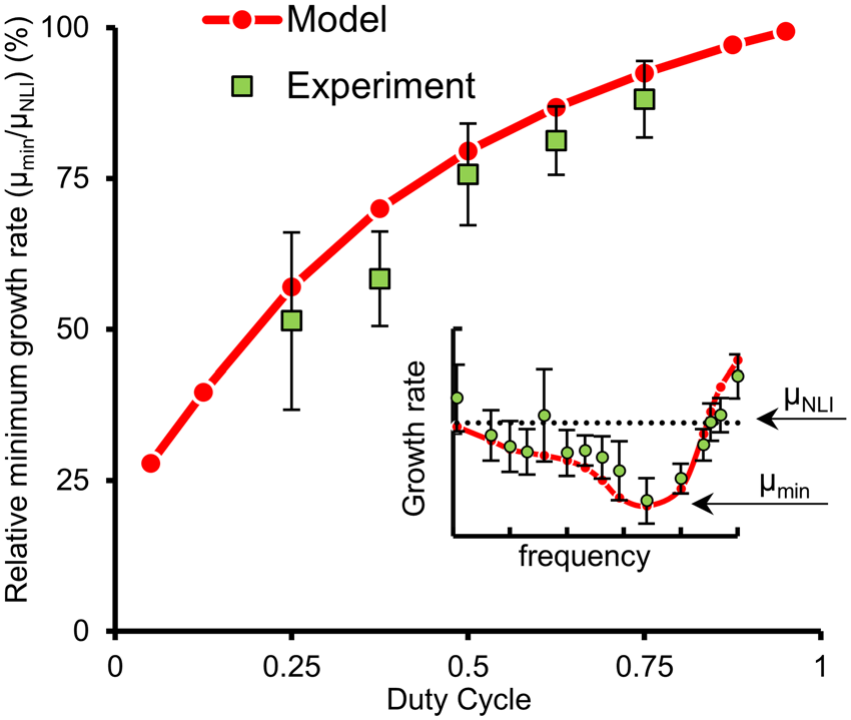
Effect of duty cycle on relative minimum growth rate. The minimum growth rate is expressed as a percentage of the no-light-integration growth rate. The data represents the mean and standard deviation of three well plates each with four technical replicates per condition, for a total of twelve data points, except for the 0.625 duty cycle case, which is limited to two well plates of four replicates.

The severity of the penalty decreases with increasing duty cycle (Fig. 3). Specifically, the minimum growth rate is approximately 50, 60, 70, 80 and 90 percent of the no-light-integration case, for duty cycles of 0.25, 0.375, 0.5, 0.625 and 0.75, respectively. In addition, results from the model are included to extend the range of duty cycles studied. The model trend is non-linear, where the penalty becomes increasingly severe with decreasing duty cycle.

### Mixotrophy tempers the fluctuating light penalty

**Figure 4:**
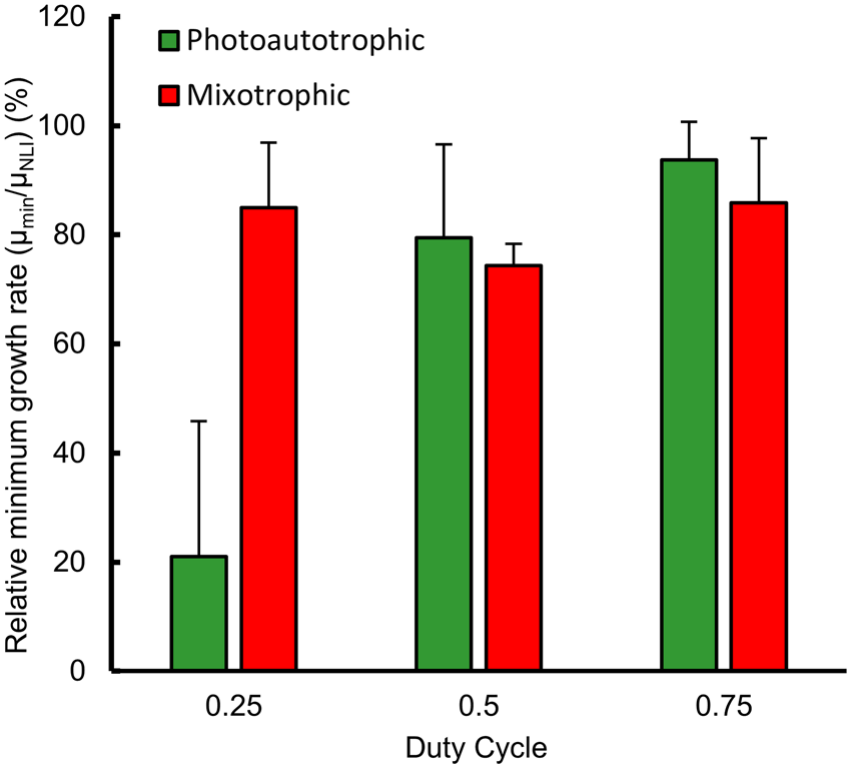
Effect of mixotrophic growth on the relative minimum growth rate for the organism *Chlamydomonas Reinhardtii*. The minimum growth rate is expressed as a percentage of the no-light-integration growth rate. Data represents the mean and standard deviation of two experiments, each with four technical replicates.

In addition to photoautotrophic growth, where organisms use light energy to reduce CO_2_ into organic forms, some photosynthetic organisms can grow photoheterotrophically, where light is used in conjunction with an organic carbon source, or heterotrophically where an organic carbon source is used as energy. *Chlamydomonas reinhardtii* wild type was used since it can grow mixotrophically, having the ability to choose between the three previously mentioned growth modes^58^. To study the effect of photoheterotrophy on the fluctuating light penalty, we grew *Chlamydomonas* in Sueoka’s high salt medium with (HSA) and without acetate (HS). In the presence of acetate (mixotrophic), the penalty is substantially decreased for a 0.25 duty cycle (Fig. 4), where the growth rate is 20% the no-light-integration case under photoautotrophic growth and 85% under photoheterotrophic growth (P ≈ 10^−5^). In comparison with *Synechococcus* (Fig. 3), *Chlamydomonas* exhibits a slightly more severe penalty for a 0.25 duty cycle, but a comparable penalty at larger duty cycles.

**Figure 5:**
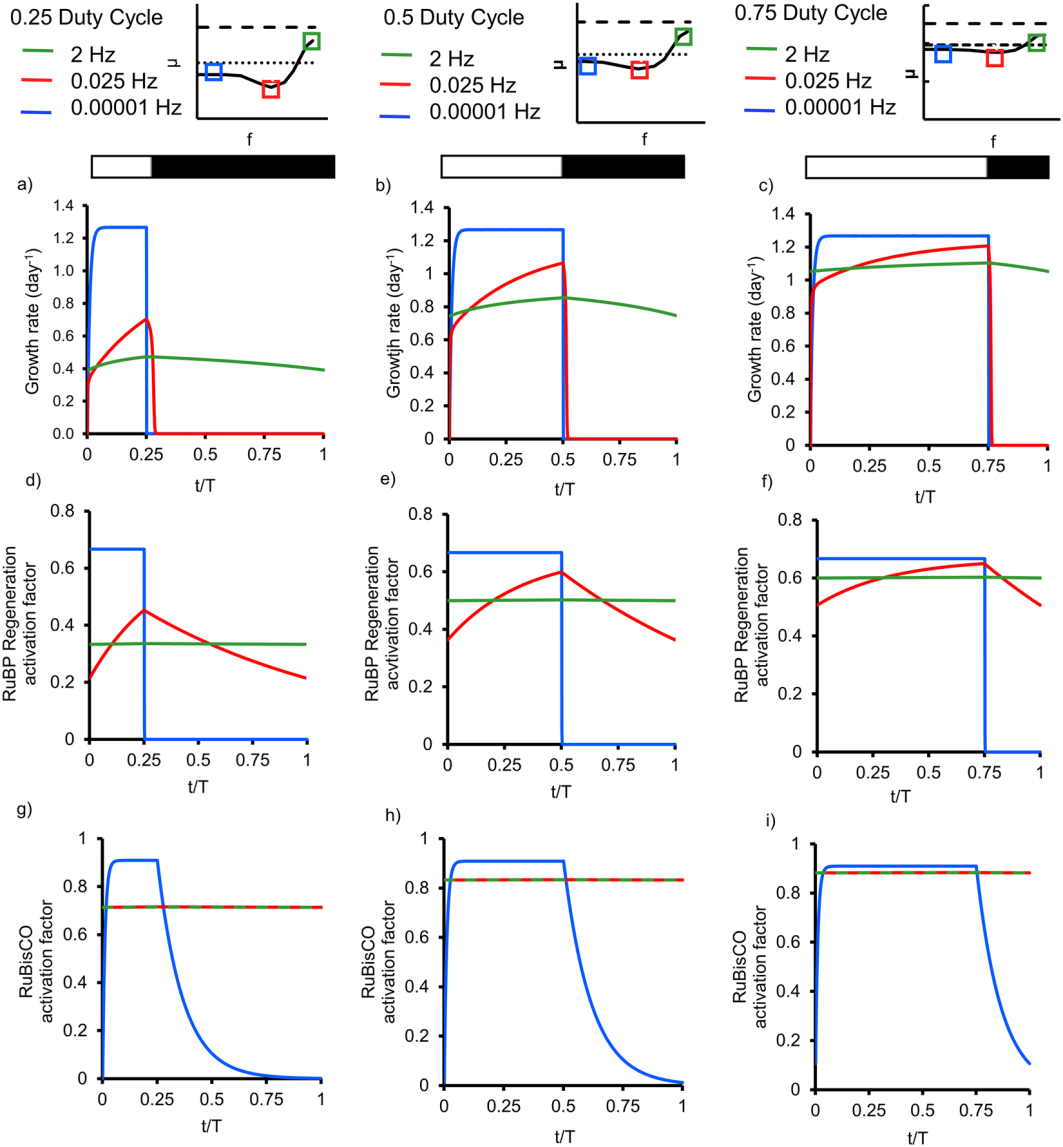
Effect of duty cycle and frequency on the transient behaviour of photosynthetic growth, based on mathematical modeling. Instantaneous growth rates (**a**–**c**), RuBP regeneration activation factor (**d**–**f**) and RuBisCO activation factor (**g**–**i**) and for duty cycles of 0.25 (**a**,**d**,**g**), 0.5 (**b**,**e**,**h**), and 0.75 (**c**,**f**,**i**), for frequencies matching continuous light, frequency which incurred a penalty and low frequency matching the growth integration.

To gain insight into the transient behaviour of photosynthesis in fluctuating light, we apply the model to track the instantaneous growth (Fig. 5a–c), the RuBP regeneration activation factor (d, e, f) and the RuBisCO activation factor (g, h, i) over one period. These activations factors represent the relative activity of the two light regulated aspects of the Calvin cycle (RuBP regeneration and RuBisCO activity). In continuous darkness, the factors are 0, and under continuous light the factors are given by the equilibrium value, which can be calculated as *K* = ${{k}}_{{on}}/({k}_{{on}}+{k}_{{off}})$. These variables are shown for duty cycles of 0.25, 0.5 and 0.75 at high frequency, where growth is above the no-light-integration case, moderate frequency where growth is below the no-light-integration case and at low frequency, where growth matches the no-light-integration case. The response for instantaneous growth and RuBP regeneration follow a square wave for the lowest frequency, since the rates are multiple orders of magnitude faster than the fluctuation frequency. Similarly, the RuBisCO activation factor increases nearly instantaneously in the light phase, then gradually deactivates in the dark phase.

For intermediate frequencies, corresponding to cases below the growth integration line, the instantaneous growth does not reach the growth achieved under the low frequency case, thus for the bulk of the light phase photosynthetic growth is impaired, to some degree. The decreased instantaneous growth rate is caused by the regulation of the Calvin cycle, particularly RuBP regeneration, being partially active for most, if not all, of the period (Fig. 5d–f).

Similar to the case of the intermediate frequencies, under high frequency flashing light, the instantaneous growth rate does not reach the growth achieved under peak irradiance value. However, the presence of growth during the dark phase compensates for the slower growth during the light phase, allowing for full-light-integration. Both the RuBisCO activation factor at frequencies of 0.025 and 2 Hz and the RuBP regeneration at frequencies of 2 Hz are relatively constant over a period, since the light fluctuations are over two orders of magnitude faster than the rate constants. However, the activation factors are below the equilibrium activation factors (0.666 for RuBP regeneration and 0.91 for RuBisCO activation, (Fig. 5d–i blue lines corresponding to 0.00001 Hz)), suggesting that the activation kinetics still impose a penalty at higher frequencies.

### The mismatch between reaction rates compensates for the regulation of the Calvin cycle at high frequencies

**Figure 6:**
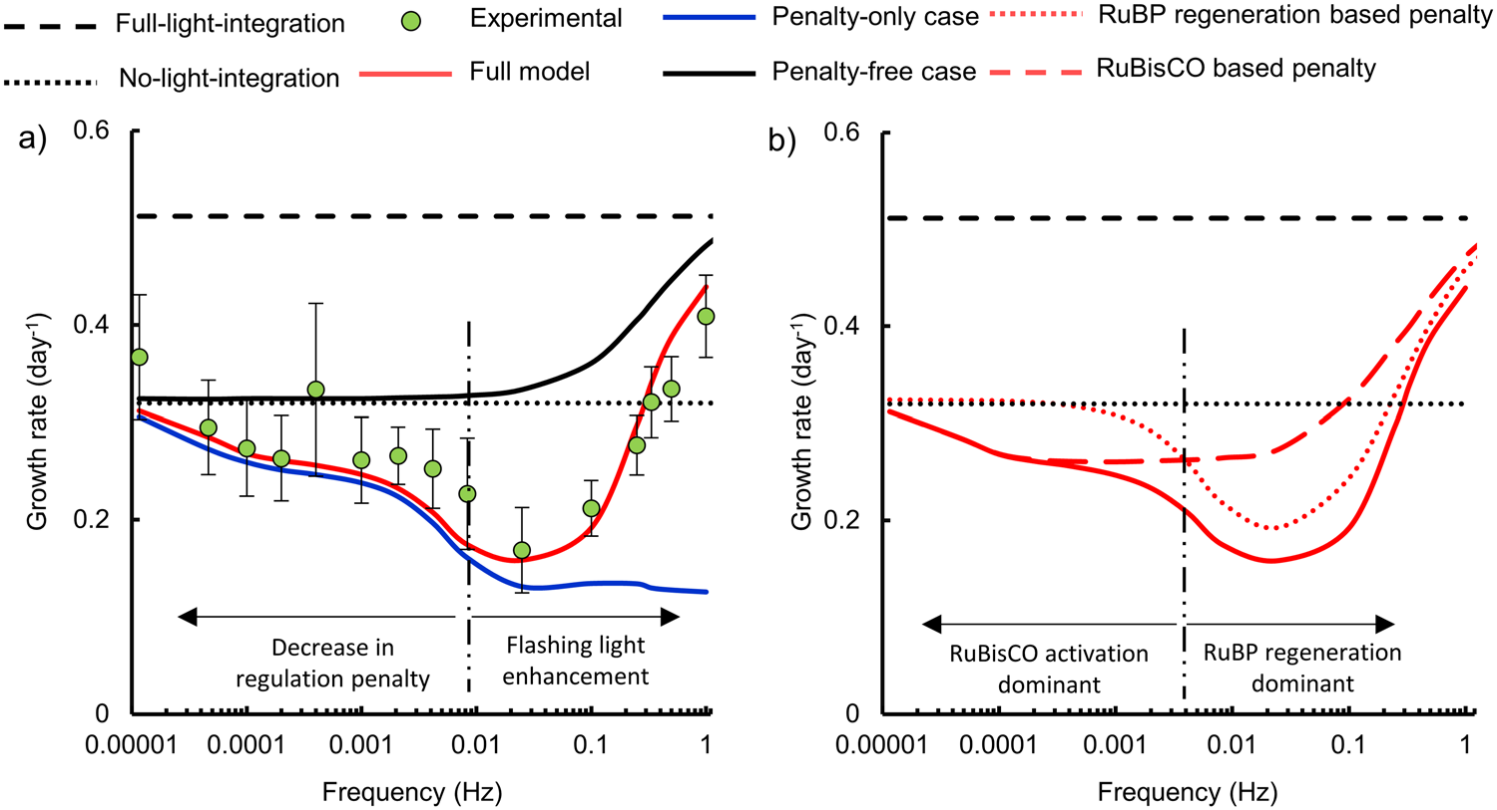
Relative contributions of (i) the conventional flashing light effect, (ii) RuBisCO regulation and (iii) RuBP regeneration on photosynthetic growth under fluctuating light, interpreted using two special cases of the mathematical model. Data corresponds to a 0.25 duty cycle. (**a**) Full model and experimental data plotted with two special cases: (i) a model free of the flashing light penalty and (ii) a model which only has the flashing light penalty and no benefit of the flashing light effect (**b**) Full model and two special cases which each have only one regulatory mechanism, either RuBisCO activation or RuBP regeneration.

The partial activation state of the Calvin cycle at high frequencies motivates reconsidering the role of the conventional flashing light effect, i.e. growth bound between a high and a low frequency limit. In Fig. 6a, the full model presented here is deconstructed into two special cases: a penalty-free case and a penalty-only case, for a 0.25 duty cycle. The penalty-free case shown is the conventional two-state model^19^ which assumes that the Calvin cycle responds instantaneously to a change in irradiance. In contrast, the penalty-only case assumes that there is no mismatch in reaction rates and by extension no growth during the dark phase, but includes the lag times for the Calvin cycle.

The penalty-free case predicts a higher growth rate than both the experimental data and the complete model for all frequencies. This discrepancy suggests that the regulation of the Calvin cycle decreases growth for all frequencies above a certain threshold, or in other words acts like a low-pass filter. Moreover, the penalty-only case shows decreasing growth with increasing frequency, affirming that growth is increasingly penalized with increasing light fluctuation frequency. The full model generally follows the penalty-only case until a frequency of roughly 0.01 Hz, where the full model begins to increase. Similarly, the penalty-free case begins to increase above the no-light-integration line at 0.01 Hz. This comparison between the full model and special cases (Fig. 6a) indicates that the slow kinetics of RuBisCO compensates for the light dependent regulation of the Calvin cycle at high frequencies.

### RuBP regeneration regulation is the dominant source of the penalty

The relative contributions of RuBisCO regulation and RuBP regeneration are shown by comparing the full model to the special cases which are penalized by either RuBisCO activation or RuBP regeneration, as opposed to both (Fig. 6b). The 0.25 duty cycle is shown as an example, since it is the case where the penalty is strongest. RuBisCO activation is the dominant source of the penalty for frequencies below 0.004 Hz, whereas RuBP regeneration imposes a penalty for frequencies above 0.004 Hz. The penalty associated with RuBisCO activation is weak, since the activation rate for RuBisCO is roughly an order of magnitude higher than the deactivation rate. In contrast, RuBP regeneration imposes a severe penalty since the activation rate is only double the deactivation rate. In addition, a shoulder is present at a frequency of roughly 0.004 Hz, where the RuBP regeneration penalty begins to increase with increasing frequency. The presence of a dip, shoulder and gradual increase is due to the combination of the two distinct regulatory mechanisms.

## Conclusion

This work presents evidence that the regulation of photosynthesis in fluctuating light has an adverse effect on photoautotrophic growth, particularly strong in a range around 0.01–0.1 Hz - a range that is typical of nature, agriculture, and industry. We postulate that the light dependent regulation of the Calvin cycle is the biological process responsible for impeding growth. As such, under photoheterotrophic conditions, the penalty is substantially reduced, highlighting the role of the Calvin cycle. The ability of photoheterotrophic growth to reduce the penalty attests to the role of the regulation of the Calvin Cycle in penalizing photosynthetic growth. Under photoheterotrophic growth, organic carbon is acquired instead of CO_2_, reducing the need for the Calvin cycle^59,60^. Non-photochemical quenching (NPQ) is also regulated by irradiance, thus in principle could be a source of a penalty^61^. However, the required irradiance to induce NPQ is on the order of hundreds to thousands of µmol·m^−2^ s^−1^ ^61–63^. Moreover, in the case of cyanobacteria, blue light is required to induce NPQ via photoprotection from orange carotenoid proteins^63^. Thus 200 µmol·m^−2^ s^−1^ of white light is not expected to produce a substantial amount of NPQ. The findings here, demonstrating a delay in CO_2_ fixation when transitioning from dark to light, complement the dynamic behaviour of NPQ, a delay in CO_2_ fixation when transitioning from excess light to moderate light.

The penalty is demonstrated here for organisms that use the Calvin cycle for carbon fixation. We expect that similar penalties may exist in other organisms where light regulation plays a role, for instance in regulating the reverse Krebs cycle of green sulfur bacteria. Purple non-sulfur bacteria, which only have a single photosystem^64,65^ likely exhibit a penalty since the Calvin cycle can be indirectly regulated by light through the redox state of the cell. Moreover, accumulation of RuBP can increase transcription of proteins relevant to the regulation of the Calvin cycle^66^. Considering that the Calvin cycle is tightly regulated in multiple organisms, it is likely that a similar penalty will exist in purple non-sulfur bacteria.

Two facets of the Calvin cycle are presented as a source of the penalty, (i) regeneration of RuBP and (ii) the activation state of RuBisCO, as shown by our mathematical model. There is a distinct penalty strongest at approximately 0.02 Hz, attributed to RuBP regeneration regulation, while at lower frequencies ranging from 0.001 to 0.0002 Hz there is a subtle penalty due to RuBisCO regulation. Though *Synechococcus elongatus* and *Chlamydomonas reinhardtii* may not express RuBisCO activase specifically, the catalytic state of RuBisCO is maintained in the light by enzymes which operate in a similar fashion^67^. Moreover, our numerical model clarifies the transient nature of photosynthesis in fluctuating light. Specifically, the Calvin cycle is not fully active during the light phase, which in turn penalizes growth in fluctuating light. Furthermore, we expand the scope of the traditional flashing light effect from enabling improved growth above the no-light-integration case to allowing for the organism to compensate for the regulation of the Calvin cycle. This is noteworthy, in that RuBisCO’s slow kinetics are considered to be a disadvantage^18^, however, our results suggest that these slow kinetics are in some sense desirable since they allow a photosynthetic organism to compensate for delayed activation of the Calvin cycle.

Our data suggests that the causes of light fluctuations could be indirectly considered as stressors, should they force fluctuations into frequencies in the penalized range. Specifically, changes in the physical environment such variations in wind and natural convection driven mixing coupled to increases in turbidity could reduce photoautotrophic growth. Regarding climate change, increases in temperature are having an impact on circulation in bodies of water^68–70^, and therefore the second order behaviour of photoautotrophic growth should be considered when forecasting the impacts of climate change. Furthermore, the majority of controlled experiments have been performed under steady state irradiance conditions^71^, and do not capture any interactive effects between environmental drivers and fluctuating light. Since fluctuating light imparts a penalty on growth, experiments with continuous irradiance could be underestimating the harm imposed by environmental stressors, such as CO_2_ acidification stress or nutrient limitation^5^. First, CO_2_ acidification stress can be more severe under a higher intensity light in marine species^61^. Second, light and CO_2_ can be seen as aiding resources^72^, due to their role in supplying energy to the cell, an organism in fluctuating light may be more sensitive to nutrient limitation. Causes of fluctuating irradiance should be considered when assessing ecosystems, either due to a direct penalty or in combination with various stressors.

The penalty is present in both a cyanobacteria and a eukaryotic algae under photoautotrophic growth. However, under mixotrophic conditions (the ability to adopt either photoautotrophic, photoheterotrophic or chemoheterotrophic growth), *Chlamydomonas* exhibits little to no penalty compared with the no-light-integration case and as duty cycles are changed. The ability of mixotrophy to alleviate the flashing light penalty broadens the role of mixotrophy from the ability to thrive in low light and CO_2_ to the ability to thrive where light is intermittent. It is important to note that alternating between photoautotrophic, photoheterotrophic and heterotrophic growth requires some adjustment in metabolism and transcription^36,73^, which could incur an additional, small penalty. Mixotrophy is common in organisms evolved to live in lower light or CO_2_^74^. Our results indicate a broadened role of mixotrophy, enabling growth where light is intermittent.

Lastly, these results are pertinent to biotechnology. The presence of a penalty in *Chlamydomonas* suggests a similar behaviour may exist in higher plants (by extension agriculture), since they share similar carbon fixation machinery^75^. To improve agricultural yields, researchers often suggest re-engineering RuBisCO to improve crop performance^76^. Though re-engineering of RuBisCO could be effective, our data highlight that increasing the activation rate of the Calvin cycle would improve growth through better light utilization. Similarly, researchers have recently demonstrated that an accelerated recovery from NPQ can increase yields in tobacco^77^. Photoprotection in the form of NPQ was shown to occur for an irradiance intensity of 2000 µmol·m^−2^ s^−1^. As the light decreases to 200 µmol·m^−2^ s^−1^ organisms continue to exhibit NPQ for a short period of time which reduces light harvesting and in turn CO_2_ fixation. Our findings complement this research, by further demonstrating the benefit of improving the dynamics of the regulation of photosynthesis. Lastly, current models for predicting photobioreactor performance are bound between the full-light-integration and no-light-integration behaviours. As such these models do not capture the photosynthetic penalty associated with fluctuating light that is inherent to most operations and particularly at high densities.

## Methods

### Cell culture conditions

*Synechococcus elongatus* PCC7942 T2SEΩ (provided by Professor Rakefet Schwarz, Bar-Ilan University, Israel) was cultured at 28 C under continuous irradiance of 45 µmol·m^−2^ s^−1^ and room CO_2_ (≈600 ppm) for long term storage^78^. Approximately 48 hours prior to experimentation, cells were transferred to a 1% CO_2_ environment. Cells were cultured in a double concentration BG-11 media (PhytoTechnology Laboratories, Shawnee Mission, Kansas, USA), which was pH buffered to 7.5 with 20 mM HEPES (Sigma Aldrich, Oakville, Ontario Canada). Cell culture media was sterilized using 0.22 µm vacuum filtration units. This modified strain is resistant to kanamycin.

Wild-type *Chlamydomonas reinhardtii (CC-124)*, was obtained from the Chlamydomonas Resource Centre (University of Minnesota, St. Paul, Minnesota). The organism was culture with either Sueoka’s high salt medium with (HSA) and without acetate (HS), pH buffered to 7 with a 20 mM concentration of MOPS. *Chlamydomonas reinhardtii* cells were maintained at 25 C under a continuous irradiance of µmol·m^−2^ s^−1^ and room CO_2_ (≈600 ppm), for long term storage. Approximately 48 hours prior to experimentation, cells were transferred to a 1% CO_2_ environment.

### Experimental apparatus

Spatial control of irradiance across a 384-well plate was achieved using an LCD projector (EPSON EX3220 SVGA 3LCD), shown in Fig. 1a and a sample of the resulting irradiance pattern shown in Fig. 1b. The various irradiances cases were achieved by creating a 24 hour video file using a MATLAB script (included as supplemental). The resulting video was then played using VLC media player (VideoLAN, Paris, France). The frequency and duty cycle layouts are shown in Fig. S1a,b, respectively. In addition, the spectral profile is included as Fig. S2. The video file was manually aligned to the well plate, and continued alignment was ensured by mounting both the projector and well plate to an optical bread board. The well plate was incubated at 1% CO_2_ by enclosing the plate in a custom made transparent acrylic chamber, which was fed with a 1% CO_2_ gas mixture. Temperature was maintained at 28 C for *Synechococcus elongatus* and 25 C for *Chlamydomonas reinhardtii* using resistive heaters and a thermostat.

### Experimental protocol

Approximately 48 hours prior to being transferred to the 384-well plate, *S. elongatus* was re-suspended to an optical density of 0.05 (measured in a 1 cm cuvette) and cultured at 1% CO_2_ under an irradiance of 80 µmol·m^−2^ s^−1^, supplied by the projector used during the experiment. The CO_2_ was monitored using a NIR CO_2_ sensor (CO2meter). This was done to acclimate the cells to both the CO_2_ concentration and irradiance spectral profile. A 1% CO_2_ was chosen increase the transport into the chambers (details in SI).

For the purposes of screening the effect of irradiance frequency on growth, microalgae were diluted to an optical density of 0.05 (measured in a 1 cm cuvette), then loaded into a 384-well plate. Each chamber of an opaque 384-well plate received 50 µL of cell suspension. During culturing, the plate was sealed using a breathable film (Double Skin, 4titude). The well plate was mounted to an optical bread board located below a projector and aligned to a video file to create the various light conditions and to ensure continued alignment during the experiment.

During measurement, the breathable film was replaced with an optical grade plate sealing film (VIEWseal, Greiner). Optical density in the 384-well plate was measured using a BMG PheraStar plate reader. Prior to measurement, the plates were vortex mixed then centrifuged briefly (30 s) at approximately 130 g’s to ensure well dispersed cells and to eliminate bubbles. After the measurement phase, the optical film was replaced with a new breathable film and the well plate was re-mounted to the experimental setup.

### Growth under continuous irradiance

The continuous irradiance data was included on the same well-plate as the growth under fluctuating irradiance. For *Synechococcus elongatus*, exponential growth was maintained over the course of the two day experiment as shown by the high light case included in Supplementary Fig. S2. For *Chlamydomonas reinhardtii*, experiments were run for three days, however growth rates over either two or three days were used, due to the vastly different growth rates for the various conditions.

## Electronic supplementary material

Supplementary Information

Supplementary Dataset 2

Supplementary Dataset 3

Supplementary Dataset 1

Supplementary Dataset 4

Supplementary Dataset 5

Supplementary Dataset 6

Supplementary Dataset 7

Supplementary Dataset 8

Supplementary Dataset 9

Supplementary Dataset 10

Supplementary Dataset 11

Supplementary Dataset 12

Supplementary Dataset 13

Supplementary Dataset 14

Supplementary Dataset 15
