# Supplementary material for: A penalty on photosynthetic growth in fluctuating light
Source: Sci Rep. 2017 Oct 2;7:12513. doi: 10.1038/s41598-017-12923-1 (PMC5624943; doi:10.1038/s41598-017-12923-1)
Supplement: Supplementary file 1 — Supplementary Information [file 41598_2017_12923_MOESM1_ESM.pdf]

A Penalty on photosynthetic growth in fluctuating light

Author List:

Percival J Graham, PhD, University of Toronto Mechanical and Industrial Engineering
Brian Nguyen, JD, University of Toronto Mechanical and Industrial Engineering
Thomas Burdyny, MASc, University of Toronto Mechanical and Industrial Engineering
David Sinton, PhD, University of Toronto Mechanical and Industrial Engineering

Corresponding Author:

David Sinton,
Phone: (416) 978-1623 | Fax: (416) 978-7753 | Email: sinton@mie.utoronto.ca
Dept. of Mechanical & Industrial Engineering
5 King's College Road,
Toronto, ON Canada, M5S 3G8

Supplementary Information

Contents

1. **Frequency and Duty cycle layouts**
2. **Continued exponential growth**
3. **Carbon dioxide concentration in microwells**
4. **Irradiance spectral distribution**
5. **Schematic representation of the mathematical model**
6. **Nomenclature of Mathematical Model**
7. **Details of Mathematical Model**
8. **Details regarding parameters**
9. **References**

1. Frequency and Duty cycle layouts

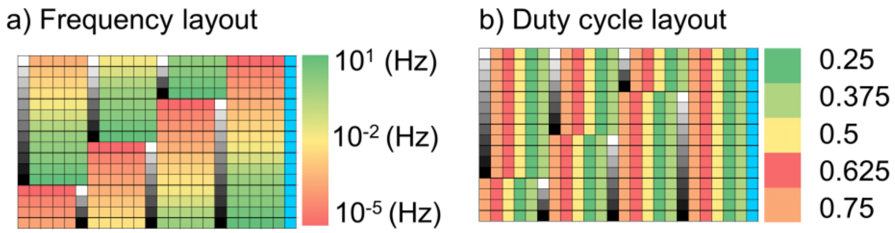

Figure S1: (a) Layout of frequencies and (b) duty cycles used, the colours indicate a log scale in the case of frequency and linear for duty cycle, grayscale colouring indicates continuous irradiance cases and light blue indicates empty chambers used as blanks.

2. Continued exponential growth

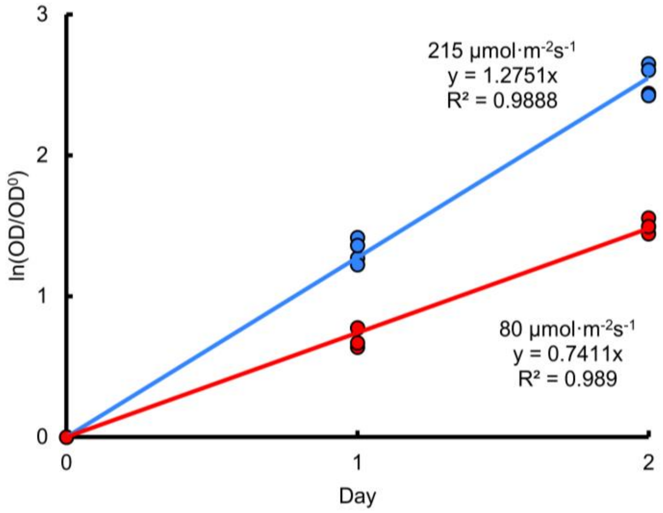

Figure S2. Continued exponential growth over two days for the fastest growing case of 215 µmol·m^{-2}s^{-1} and the intermediate irradiance of 80 µmol·m^{-2}s^{-1}, for *Synechococcus elongatus.*

3. Carbon dioxide concentration in the microwells

The carbon dioxide concentration around the well plate was maintained at 1% during the course of the experiment by enclosing the well plate in a clear acrylic box and flushing the box with a 1% CO_2-in-air gas mixture. This concentration was used to ensure adequate CO_2 transport through the breathable film and into the culture. Transport through both the breathable film and the cell suspension can be understood through Fick's Law.

$$J = D\frac{\partial c}{\partial x}$$

Where J is the flux, D the diffusivity, c the concentration and x distance. We can calculate the flux required based on (i) the CO_2 rate of a cell (roughly 5 x 10^{-18} mol/cell*s)[1] and (ii) assuming a relatively dense cell suspension of 10^7 cells/ml with (iii) 50 µL of cell suspension per well and each well has a surface of (9mm^2). The flux is then roughly 3 x 10^{-7} mol/m^{2}s. To achieve such a flux, the concentration gradient across the membrane (dc/dx), assuming a diffusivity through the membrane of 5 x 10^{-8} m^2/s^2, would need to be roughly 10^{-2} mol/m^3 which would reduce the CO_2 concentration by less than 10%. Moreover, continued exponential growth suggests that the algae are not CO_2 limited, since growth did not decrease substantially as the transport requirement increased.

4. Irradiance Spectral Distribution

A large portion of the irradiance spectrum (figure S3) is in the range of 500-600nm, where the absorption of *Synechoccus elongatus* ranges from 40-60%. This may suggest that light is not well absorbed by *Synechoccus elongatus*, however, light is sufficiently absorbed to drive growth, as seen by the saturating relationship between light intensity and growth rate in Figure 2a. Specifically, the cells are no longer light-limited for irradiances over 150 $\mu mol \cdot m^{-2}s^{-1}$, seen by the levelling off in Figure 2a. Both photosystems II and I are excited under this irradiance spectrum, since the phycobilisomes absorb light in the 550-650nm range and transfer this light energy to either photosystem. [3,4]

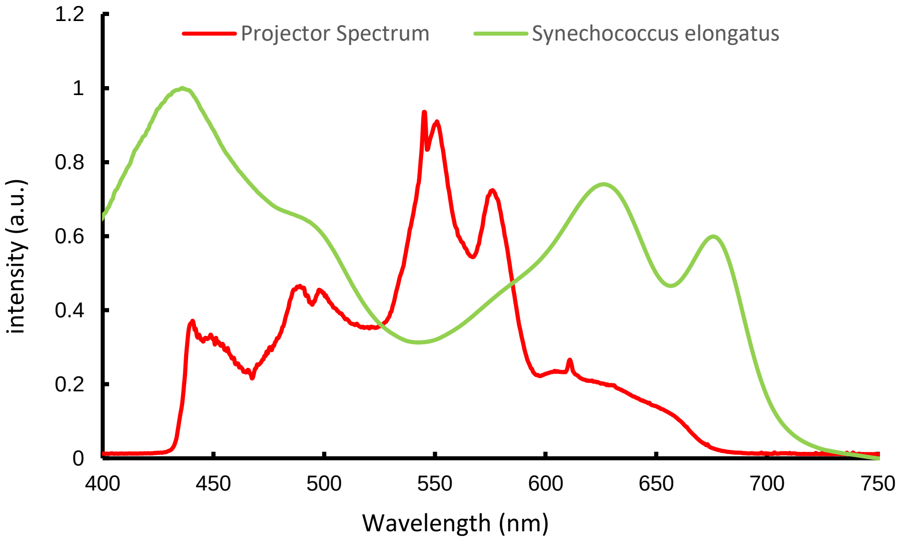

Figure S3: Irradiance Spectrum of the light source used here and the absorption spectrum of *Synechococcus elongatus*. Measured with an integrating sphere and spectrometer.

5. Schematic representation of the mathematical model

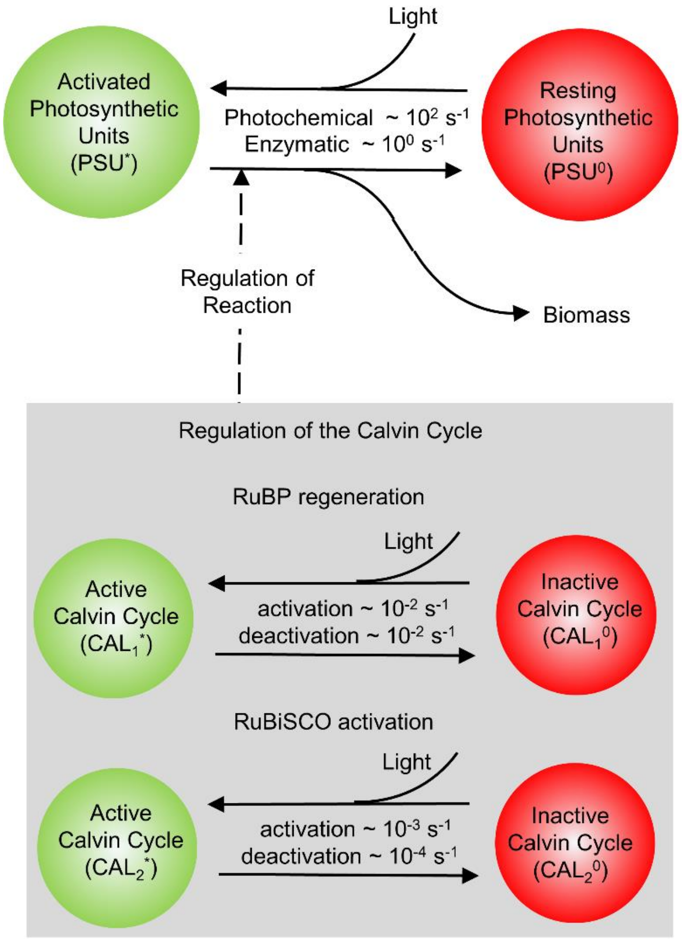

Figure S4: computational model used. The figure from the main text is repeated for convenience and the associated equations are derived in full.

6. Nomenclature of Mathematical Model

I: Irradiance intensity (measured)

PSU^0: Resting Photosynthetic Unit

PSU^*: Activated Photosynthetic Unit

CAL_1^0 : Inactive Calvin cycle for RuBP regeneration

CAL_1^* : Active Calvin cycle for RuBP regeneration

CAL_2^0 : Inactive Calvin cycle for RuBisCO

CAL_2^* : Active Calvin cycle for RuBisCO

k_{cal1ON} : activation rate of RuBP regeneration (fitted manually)

$k_{cal1OFF}$: deactivation rate of RuBP regeneration (fitted manually)

k_{cal2ON} : activation rate of RuBisCO (fitted manually)

$k_{cal2OFF}$: deactivation rate of RuBisCO (fitted manually)

x^* : Fraction of activated PSUs (determined from the model)

y^* : Fraction of active RuBP regeneration (determined from the model)

z^* : Fraction of active RuBisCO (determined from the model)

x_{ss}^* : Fraction of activated PSUs at steady state (determined from the model at steady state)

y_{ss}^* : Fraction of active RuBP regeneration at steady state (determined from solving the model at steady state)

z_{ss}^* : Fraction of active RuBisCO at steady state (determined from solving the model at steady state)

R : maximum rate of deactivation of activated photosynthetic units

K : half saturation constant

k_a : light harvesting constant

P_m : maximum growth rate (determined by fitting steady state solution)

B : maximum rate of deactivation of activated photosynthetic units, relative to the total amount of photosynthetic units. (fitted manually)

α : ratio between enzymatic and photochemical rates (determined by fitting steady state solution)

κ : half saturation constant, relative to the total amount of photosynthetic units. (fitted)

7. Details of Mathematical Model

This section describes the equations used in the model and shows the steps progressing from the formulation of the model, to non-dimensionalization of the model, to solving the systems in both steady state and for fluctuating light. Equations (1-6) represent the model scheme, equations (7), (8) and (11) are the non-dimensional equations derived from equations (1-6). Equation 12 is used to extract data from the continuous light curve.

For The PSUs

$$\frac{dPSU^0}{dt} = -k_a IPSU^0 + \frac{CAL_1{}^*}{CAL_1{}^* + CAL_1{}^0} \frac{CAL_2{}^*}{CAL_2{}^* + CAL_2{}^0} \frac{R\ PSU^*}{K_S + PSU^*} \tag{1}$$

$$\frac{dPSU^*}{dt} = k_a IPSU^0 - \frac{CAL_1{}^*}{CAL_1{}^* + CAL_1{}^0} \frac{CAL_2{}^*}{CAL_2{}^* + CAL_2{}^0} \frac{R\ PSU^*}{K_S + PSU^*} \tag{2}$$

For the degree of Calvin cycle activation,

$$\frac{dCAL_1{}^0}{dt} = -k_{cal1ON} CAL^0 + k_{cal1OFF} CAL^* \tag{3}$$

$$\frac{dCAL_1{}^*}{dt} = k_{cal1ON} CAL^0 - k_{cal1OFF} CAL^* \tag{4}$$

$$\frac{dCAL_2{}^0}{dt} = -k_{cal2ON} CAL^0 + k_{cal2OFF} CAL^* \tag{5}$$

$$\frac{dCAL_2{}^*}{dt} = k_{cal2ON} CAL^0 - k_{cal2OFF} CAL^* \tag{6}$$

Where k_{cal1ON} *and* k_{cal2ON} are 0 in the dark.

Taking into account that the sum of the active and inactive Calvin cycle should remain constant, we non-dimensionalize the concentration of active Calvin Cycle molecules as,

$$\frac{dy^*}{dt} = k_{cal1ON} (1 - y^*) - k_{cal1OFF} y^* \tag{7}$$

Where,

$$y^* = \frac{CAL_1{}^*}{CAL_1{}^* + CAL_1{}^0}$$

$$\frac{dz^*}{dt} = k_{cal2ON} (1 - z^*) - k_{cal2OFF} z^* \tag{8}$$

Where,

$$z^* = \frac{CAL_2{}^*}{CAL_2{}^* + CAL_2{}^0}$$

Under continuous irradiance the activity of the Calvin cycle can be determined from the steady state solution of equation (5).

$$0 = k_{cal1ON}\,(1 - y^*_{ss}) - k_{cal1OFF}\,y^*_{ss}$$

$$0 = k_{cal2ON}\,(1 - z^*_{ss}) - k_{cal2OFF}\,z^*_{ss}$$

Giving the following for the active fraction

$$y^*_{ss} = \frac{K_{Calvin1}}{K_{Calvin1} + 1} \quad (9)$$

$$z^*_{ss} = \frac{K_{Calvin2}}{K_{Calvin2} + 1} \quad (10)$$

Where,

$$K_{Calvin1} = \frac{k_{cal1ON}}{k_{cal1OFF}}$$

$$K_{Calvin2} = \frac{k_{cal2ON}}{k_{cal2OFF}}$$

Taking into account that the sum of the active and inactive photosynthetic units should remain constant, we non-dimensionalize the concentration of active photosynthetic units as,

$$\frac{dx^*}{dt} = \beta\left(\frac{I}{\alpha}\left(\frac{K_{CAL1}}{K_{CAL1}+1}\right)\left(\frac{K_{CAL2}}{K_{CAL2}+1}\right)(1 - x^*) - y^* z^* \frac{x^*}{\kappa + x^*}\right) \quad (11)$$

Where,

$$\beta = \frac{R}{PSU^0 + PSU^*},\ \alpha = \frac{R}{k_a(PSU^0 + PSU^*)}\frac{K_{CAL1}}{K_{CAL1}+1}\frac{K_{CAL2}}{K_{CAL2}+1},\ \kappa = \frac{K_S}{PSU^0 + PSU^*},\ x^* = \frac{PSU^*}{PSU^0 + PSU^*}$$

At steady state conditions we get,

$$0 = \beta\left(\frac{I}{\alpha}\left(\frac{K_{CAL1}}{K_{CAL1}+1}\right)\left(\frac{K_{CAL2}}{K_{CAL2}+1}\right)(1 - x^*_{ss}) - y^*_{ss} z^*_{ss} \frac{x^*_{ss}}{\kappa + x^*_{ss}}\right)$$

Substituting the previously determined steady state values y_{zz} *and* z_{ss} *we obtain,*

$$0 = \beta\left(\frac{I}{\alpha}\left(\frac{K_{CAL1}}{K_{CAL1}+1}\right)\left(\frac{K_{CAL2}}{K_{CAL2}+1}\right)(1 - x^*_{ss}) - \left(\frac{K_{CAL1}}{K_{CAL1}+1}\right)\left(\frac{K_{CAL2}}{K_{CAL2}+1}\right)\frac{x^*_{ss}}{\kappa + x^*_{ss}}\right)$$

Which simplifies to

$$0 = \frac{I}{\alpha}(1 - x_{SS}^*) - \frac{x_{SS}^*}{\kappa + x_{SS}^*}$$

Solving for x_{ss}*,

$$0 = {x_{SS}^*}^2 + \left(\frac{\alpha}{I} + \kappa - 1\right)x_{SS}^* - \kappa$$

$$x_{SS}^* = \frac{\left(1 - \kappa - \frac{\alpha}{I}\right) \pm \sqrt{\left(1 - \kappa - \frac{\alpha}{I}\right)^2 - 4\kappa}}{2}$$

The rate of biomass accumulation is proportional to the rate at which PSUs decay from excited to unexcited,

$$\frac{P}{P_m} = y^* z^* \frac{x^*}{\kappa + x^*}$$

Where the product, $P_m y^*$, is the maximum growth rate

At steady state we obtain,

$$\frac{P}{P_m} = \frac{I}{\alpha}\left(\frac{K_{CAL1}}{K_{CAL1}+1}\right)\left(\frac{K_{CAL2}}{K_{CAL2}+1}\right)(1 - x_{SS}^*)$$

Substituting the expression for x_{ss}* we obtain,

$$P = P_m \frac{I}{2\alpha}\left(\frac{K_{CAL1}}{K_{CAL1}+1}\right)\left(\frac{K_{CAL2}}{K_{CAL2}+1}\right)\left(2 - \left(1 - \kappa - \frac{\alpha}{I}\right) - \sqrt{\left(1 - \kappa - \frac{\alpha}{I}\right)^2 - 4\kappa}\right) \quad (12)$$

Equation (12) is used to extract parameters P_m, κ and α, which are used to solve equations (7), (8) and (11) simultaneously.

Transient system

$$\frac{dy^*}{dt} = k_{cal1ON}(1 - y^*) - k_{cal1OFF}\, y^* \quad (7)$$

$$\frac{dz^*}{dt} = k_{cal2ON}(1 - z^*) - k_{cal2OFF}\, z^* \quad (8)$$

$$\frac{dx^*}{dt} = \beta\left(\frac{I}{\alpha}\left(\frac{K_{CAL1}}{K_{CAL1}+1}\right)\left(\frac{K_{CAL2}}{K_{CAL2}+1}\right)(1 - x^*) - y^* z^* \frac{x^*}{\kappa + x^*}\right) \quad (11)$$

8. Details regarding parameters

The continuous light data gave values of 141, 0.04357 and 1.425 for parameters α, κ and P_m, respectively (R^2 = 0.997). With regards to parameters relevant to fluctuating light, the parameter β, chosen here to be 2, represents the maximum rate of de-excitation of active photosynthetic units relative to the total amount of photosynthetic units in a cell. This parameter relates to the frequency required to reach full light integration, which is not the focus of this work. Values between 2-15 will have been shown to fit previous experimental data.[5,6] An approximation of β, could be the rate at which a cell can reduce carbon per photosystem. This can be approximated by considering the RuBisCO catalytic rate (3-10 s^{-1}),[7,8] the number of RubisCO active sites per carboxysome (2000-8000),[8,9] and the number of carboxysomes per cell (~4)[10] cell and the number of photosystems per cell (~96 000)[11], giving values of β of roughly 0.2-3, which is reasonable in comparison to the fitted parameters. In short, the value of the parameter β agrees with previous works and is in general agreement with what would be expected based on the constituents of a photosynthetic organism.

Irradiance controls the degree of activation of the Calvin cycle by indirectly activating Rubisco Activase by increasing the amount of ATP and by promoting RuBP regeneration via redox sensing of the photosynthetic electron transport chain.[12] Precise values for relevant rate constants are sparse, since the details of the regulation of the Calvin cycle is an ongoing research topic,[7,13] activation and deactivation kinetics vary across species[14–16] and the rate constants are typically extracted by fitting complex models to CO_2 assimilation experiments. [17–19] The activation rates used in our model align well with experimental values measured for CO_2 assimilation in leaves with active RuBisCO (0.2-0.01 s^{-1})[15,17,18,20–22]. Deactivation rates for RuBP regeneration are not

explicitly available experimentally, but can be inferred to be a factor of 1-4 times slower than the activation rate based on various models fit to data.[17,18,20] The regulation of RuBisCo activity is typically one to two orders of magnitude slower than RuBP regeneration with an activation rate constant 5-10 times higher than the deactivation rate constant.[16–18,20,23]. Data for six marine diatoms and a cyanobacterial species show RuBisCO activation rates in the range of 0.01 – 0.001 s^{-1} [15,16,24]. Highlighting the kinetics related to the catalytic rate of RuBisCO in microalgae. Our fitted experimental values are in agreement with the values reported in literature.

9. References

1. Fridlyand, L., Kaplan, a & Reinhold, L. Quantitative evaluation of the role of a putative CO2-scavenging entity in the cyanobacterial CO2-concentrating mechanism. *Biosystems.* **37,** 229–38 (1996).

2. Sieben, M., Giese, H., Grosch, J.-H., Kauffmann, K. & Büchs, J. Permeability of currently available microtiter plate sealing tapes fail to fulfil the requirements for aerobic microbial cultivation. *Biotechnol. J.* **11,** 1525–1538 (2016).

3. Liu, H. *et al.* Phycobilisomes supply excitations to both photosystems in a megacomplex in cyanobacteria. *Science* **342,** 1104–7 (2013).

4. Mullineaux, C. W. Phycobilisome-reaction centre interaction in cyanobacteria. *Photosynth. Res.* **95,** 175–82 (2008).

5. Rubio Camacho, F., García Camacho, F., Fernández Sevilla, J. M., Chisti, Y. & Molina Grima, E. A mechanistic model of photosynthesis in microalgae. *Biotechnol. Bioeng.* **81,** 459–473 (2003).

6. Brindley, C., Jiménez-Ruíz, N., Acién, F. G. & Fernández-Sevilla, J. M. Light regime optimization in photobioreactors using a dynamic photosynthesis model. *Algal Res.* **16,** 399–408 (2016).

7. Hauser, T., Popilka, L., Hartl, F. U. & Hayer-Hartl, M. Role of auxiliary proteins in Rubisco biogenesis and function. *Nat. Plants* **1,** 15065 (2015).

8. Rae, B. D., Long, B. M., Badger, M. R. & Price, G. D. Functions, Compositions, and

Evolution of the Two Types of Carboxysomes: Polyhedral Microcompartments That Facilitate CO2 Fixation in Cyanobacteria and Some Proteobacteria. *Microbiol. Mol. Biol. Rev.* **77,** 357–379 (2013).

9. Iancu, C. V. *et al.* The Structure of Isolated Synechococcus Strain WH8102 Carboxysomes as Revealed by Electron Cryotomography. *J. Mol. Biol.* **372,** 764–773 (2007).

10. Chen, A. H., Afonso, B., Silver, P. a & Savage, D. F. Spatial and temporal organization of chromosome duplication and segregation in the cyanobacterium Synechococcus elongatus PCC 7942. *PLoS One* **7,** e47837 (2012).

11. Keren, N. Critical Roles of Bacterioferritins in Iron Storage and Proliferation of Cyanobacteria. *Plant Physiol.* **135,** 1666–1673 (2004).

12. Kaiser, E. *et al.* Dynamic photosynthesis in different environmental conditions. *J. Exp. Bot.* **66,** 2415–2426 (2015).

13. Bracher, A., Sharma, A., Starling-windhof, A., Hartl, F. U. & Hayer-hartl, M. Degradation of potent Rubisco inhibitor by selective sugar phosphatase. *Nat. Plants* **1,** 1–6 (2015).

14. Witzel, F., Götze, J. & Ebenhöh, O. Slow deactivation of ribulose 1,5-bisphosphate carboxylase/oxygenase elucidated by mathematical models. *FEBS J.* **277,** 931–950 (2010).

15. Macintyre, H. L., Sharkey, T. D. & Geider, R. J. Activation and deactivation of ribulose-1 , 5-bisphosphate carboxylase / oxygenase (Rubisco) in three marine microalgae. *Photosynth. Res.* **51,** 93–106 (1997).

16. MacIntyre, H. L. & Geider, R. J. Regulation of Rubisco activity and its potential effect on

photosynthesis during mixing in a turbid estuary. *Mar. Ecol. Prog. Ser.* **144,** 247–264 (1996).

17. Pearcy, R. W., Gross, L. J. & He, D. X. An improved dynamic model of photosynthesis for estimation of carbon gain in sunfleck light regimes. *Plant, Cell Environ.* **20,** 411–424 (1997).

18. Kirschbaum, M. U. F., Küppers, M., Schneider, H., Giersch, C. & Noe, S. Modelling photosynthesis in fluctuating light with inclusion of stomatal conductance, biochemical activation and pools of key photosynthetic intermediates. *Planta* **204,** 16–26 (1998).

19. Carmo-Silva, A. E. & Salvucci, M. E. The Regulatory Properties of Rubisco Activase Differ among Species and Affect Photosynthetic Induction during Light Transitions. *Plant Physiol.* **161,** 1645–1655 (2013).

20. Tomimatsu, H. & Tang, Y. Effects of high CO2 levels on dynamic photosynthesis: carbon gain, mechanisms, and environmental interactions. *J. Plant Res.* **129,** 1–13 (2016).

21. Pearcy, R. W., Krall, J. P. & Sassenrath-Cole, G. F. in *Photosynthesis and the Environment* 321–346 (1996).

22. Yamori, W., Masumoto, C., Fukayama, H. & Makino, A. Rubisco activase is a key regulator of non-steady-state photosynthesis at any leaf temperature and, to a lesser extent, of steady-state photosynthesis at high temperature. *Plant J.* **71,** 871–880 (2012).

23. McNevin, D., von Caemmerer, S. & Farquhar, G. Determining RuBisCO activation kinetics and other rate and equilibrium constants by simultaneous multiple non-linear regression

of a kinetic model. *J. Exp. Bot.* **57,** 3883–3900 (2006).

24. Li, L. A. & Tabita, F. R. Maximum activity of recombinant ribulose 1,5-bisphosphate carboxylase/oxygenase of Anabaena sp. strain CA requires the product of the rbcX gene. *J. Bacteriol.* **179,** 3793–3796 (1997).
